# Pro-BNP versus MEDS Score in Determining the Prognosis of Sepsis Patients; a Diagnostic Accuracy Study

**Published:** 2018-01-15

**Authors:** Majid Shojaee, Saeed Safari, Anita Sabzghabaei, Mostafa Alavi-Moghaddam, Ali Arhami Dolatabadi, Hamid Kariman, Soheil Soltani

**Affiliations:** 1 Emergency Department, Imam Hossein Hospital, Shahid Beheshti University of Medical Science, Tehran, Iran.; 2 Emergency Department, Shohadaye Tajrish Hospital, Shahid Beheshti University of Medical Science, Tehran, Iran.

**Keywords:** Pro-brain natriuretic peptide, sepsis, mortality, Emergency department, dimensional measurement accuracy

## Abstract

**Introduction:**

Pro-brain natriuretic peptide (Pro-BNP) can act as an independent predictor of mortality in septic patients. This study aimed to compare the diagnostic accuracy of pro-BNP and Mortality in Emergency Department Sepsis (MEDS) score in this regard.

**Method:**

This cross-sectional study was conducted on > 14 years old sepsis patients of an emergency department (ED), during 2 years. The level of Pro-BNP and MEDS score were measured for all eligible patients and considering one month mortality as reference, screening performance characteristics of the two tests were compared using SPSS 21 and STATS 11.

**Results:**

121 patients with the mean age of 75.87±11.82 years were studied (55.4% male). 85 (70.25%) patients had moderate to high probability of mortality according to MEDS score. The mean Pro-BNP levels of survivor and non-survivor patients were 489.69 ± 327.47 and 3954.98 ± 2717.85 pg/ml, respectively (p < 0.0001). Sensitivity and specificity of Pro-BNP (in 1000 pg/ml cut off) and MEDS score (in level 3) in prediction of 1-month mortality were 93.6 (83.7-97.9), 94.8 (84.7-98.6), 65.0 (51.9-76.3), and 98.2 (89.5-99.9), respectively. Area under the ROC curve of the two tests were 97.36 (95% CI: 92.92-94.48) and 92.31 (95% CI: 86.35-96.53), respectively (p = 0.0543).

**Conclusion:**

Pro-BNP and MEDS score both have excellent diagnostic accuracy in predicting 1-month mortality of sepsis patients. However, considering the higher sensitivity as well as availability and ease of calculation, it seems that Pro-BNP can be considered an appropriate tool for screening patients with high risk of mortality following sepsis in ED.

## Introduction:

Septic response is in fact a chain of inflammatory and anti-inflammatory processes and hormone-cell reactions that manifest clinically via systemic disorders such as: shock, myocardial disorder, activation of coagulation system and disseminated endothelial injury (1-4). Based on the report of world health organization (WHO), in United States of America more than 1 million people are affected with sepsis, about half of which lose their lives and sepsis is the third cause of mortality after heart diseases and cancers (5, 6). Timely identification of patients at risk of mortality due to sepsis can be helpful in selecting type of intervention, treatment protocol, diagnostic method, and probably improve their final outcome (7-9). Mortality in Emergency Department Sepsis (MEDS) score is an acceptable scale for predicting the prognosis of sepsis, which is not commonly used nowadays due to having many variables and its calculation being time consuming (10). Currently, biomarkers such as: Pro-BNP, lactate, CRP, D-Dimer, Pro-Calcitonin and Troponin are considered for determining the prognosis of patients with sepsis (11-13). Natriuretic peptides are used in diagnosis and risk stratification of patients with acute coronary syndrome and congestive heart failure but the role of these factors in prognosis and diagnosis of patients with septic shock is still under debate (14). Natriuretic peptides play an important role in maintenance of cardiovascular homeostasis and circulating blood volume. Pro-BNP is secreted in response to stretching in the atrium or ventricle wall or due to myocardial ischemia in 2 shapes of N-terminal Pro-BNP (NT-Pro BNP) and C-terminal peptide (BNP) (15, 16). It has been shown that Pro-BNP can act as an independent predictor of mortality in patients with cardiac shock, septic shock, and severe sepsis (17-23). However, this marker has not been able to predict short-term mortality in critically ill patients hospitalized in critical care unit with hypoxic respiratory failure (24). Therefore, in search for finding an accurate as well as easy and available scale for replacing MEDS score in emergency department (ED), the present study was designed with the aim of evaluating the diagnostic value of pro-BNP in comparison with MEDS score in determining the prognosis of sepsis patients ED.

## Methods:

***Study design and setting***

The present study is a prospective cross-sectional one that was designed and performed with the aim of determining the diagnostic accuracy of Pro-BNP in predicting the prognosis of patients with sepsis in ED of Imam Hossein Hospital, Tehran, Iran, from September 2014 to March 2016. Protocol of this study was approved by the ethics committee of Shahid Beheshti University of Medical Sciences and the researchers adhered to the principles indicated in the declaration of Helsinki regarding medical ethics. Before entering the study, the patient or their relative signed an informed written consent for participating in the study. The research team did not interfere in the diagnostic and treatment processes of the patients.

***Participants***

Patients over the age of 14 years with sepsis presenting to the ED were randomly included in the study. For random sampling, cases were selected on random days (these days were randomly selected on the first day of each month). Simultaneous presence of systemic inflammatory response syndrome (SIRS) criteria and definitive evidence of infection based on American College of Chest Physicians/Society of Critical Care Medicine consensus was considered as the definition of sepsis (25). Patients with diagnosis of sepsis, severe sepsis, or septic shock were included in the study. Cases with history of proven cardiac or kidney failure, electrocardiogram (ECG) indicating new cardiac problems and children were excluded from the study.

***Data gathering***

Demographic data (age, sex), source of infection (pneumonia; urinary tract infection; digestive disease; bedsore and…), variables needed for calculation of MEDS score (10), serum level of Pro-BNP on admission as well as 1-month outcome of the patients regarding mortality were gathered for all the patients via a pre-designed checklist.

Source of infection was determined according to the final decision of the in charge physician and considering all the laboratory, imaging, and clinical evidence.

Pro-BNP measurement was done using 1cc of the patient’s venous blood sample drawn on admission for measuring other laboratory parameters ordered by the in charge physician. Blood drawing was done by a laboratory technician who was not aware of the study. Pro-BNP level measurement was done via chemilumenescence sandwich immunoassay using Elecsys 2010 kit, Roche diagnostic, Mannheim, Germany. The kit was a high-quality and rapid (taking 18 minutes) kit that finally reports Pro-BNP rate as pg/cc.

To calculate MEDS score, a medical calculator named emcalculator was used and according to the results the patients were divided into 5 groups regarding mortality risk: very low, low, moderate, high, and very high.

Finally, the mortality status of the patients 30 days after admission to ED was followed and recorded via phone calls. It should be noted that treatment of sepsis patients in the mentioned center is done according to the protocol of surviving sepsis campaign by emergency service with consultation of infection service (26). One emergency medicine resident was in charge of data gathering, calculating MEDS score and follow up of patients by phone.

***Statistical analysis***

Minimum sample size required for the present study considering 95% confidence interval, 80% power, 7% error, and 0.63 area under the receiver operating characteristic (ROC) curve of Pro-BNP (27), was calculated to be 122 cases. Data were analyzed via SPSS 21 and STATA 11 software. Qualitative data were reported as frequency and percentage, and quantitative data as mean ± standard deviation (SD). Finally, for determining the diagnostic accuracy of Pro-BNP in predicting the 1-month prognosis of sepsis patients, ROC curve was drawn and the best cut-off was calculated. In addition, sensitivity, specificity, positive and negative predictive values, and positive and negative likelihood ratios with 95% confidence interval were calculated and reported using VassarStats medical calculator. To compare the diagnostic accuracies of the 2 tests in predicting the risk of 1-month mortality in patients with sepsis presenting to the ED, comparison of area under the ROC curve was done via chi square test. In all analyses p<0.05 was considered as level of significance. Accuracy of 0.90-0.100 was considered as excellent, 0.80-0.90 as good, 0.70-0.80 as fair, 0.60-0.70 as poor, and 0.50-.60 as fail.

## Results:

***Baseline characteristics***

**Figure 1:**
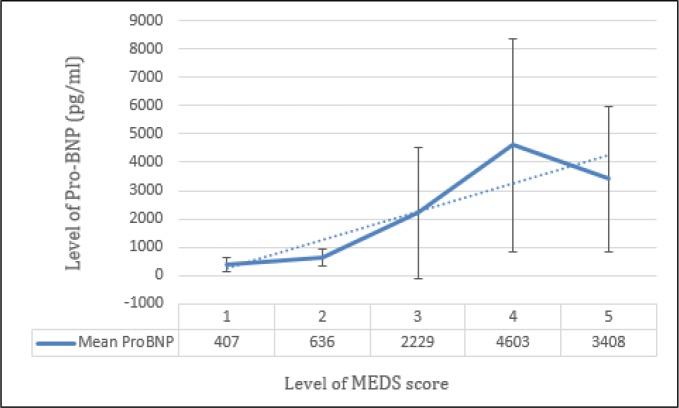
Mean and standard deviation of patients’ Pro-BNP level based on their MEDS score level.

155 patients were candidates for entering the study, 9 (5.80%) were excluded due to history of kidney failure and 7 (4.52%) due to cardiac failure. In addition, 18 (11.61%) were excluded due to missing data and problem in follow-up. Finally, 121 patients with the mean age of 75.87±11.82 (20-98) years were evaluated (55.4% male). Table 1 shows the baseline characteristics of the patients. 85 (70.25%) patients had moderate to high probability of mortality according to MEDS scale. The mean Pro-BNP levels of survivor and non-survivor patients were 489.69 ± 327.47 and 3954.98 ± 2717.85 pg/ml, respectively (p < 0.0001). Figure 1 depicts mean Pro-BNP level based on the patients’ MEDS score.

***Screening performance characteristics***

**Figure 2:**
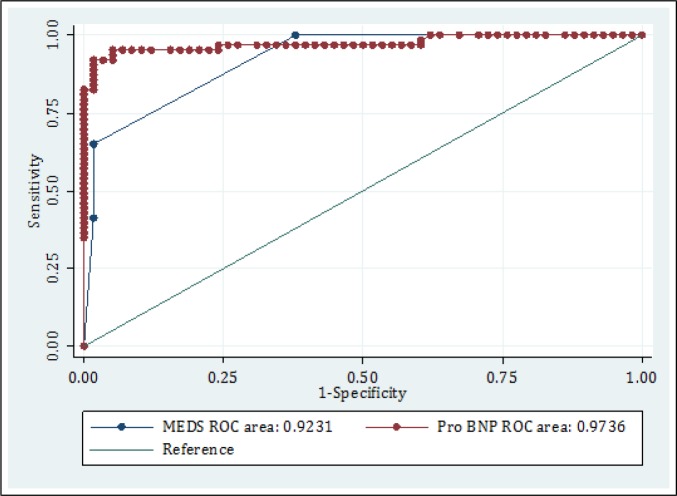
Area under the receiver operating characteristic (ROC) curve of Pro-BNP and MEDS score in determining the risk of 1-month mortality among sepsis patients in emergency department (p=0.0543).

Of all the studied patients, 63 (52.1%) died after 1 month. Area under the ROC curve of Pro-BNP and MEDS score in prediction of 1-month mortality of sepsis patients admitted to ED were 97.36 (95% CI: 92.92-94.48) and 92.31 (95% CI: 86.35-96.53), respectively (p = 0.0543, figure 2). According to the area under the ROC curve, the best cut off to predict 1-month mortality was 1000 pg/ml for Pro-BNP and level 3 for MEDS scale. Table 2 shows the screening performance characteristics of MEDS score and Pro-BNP in the mentioned cut offs.

## Discussion:

Based on the findings of the present study, Pro-BNP over 1000 pg/ml and MEDS scale both have excellent diagnostic accuracy in predicting 1-month mortality of sepsis patients. However, considering the higher sensitivity of Pro-BNP (93.6 vs. 65.0) as well as availability and ease of calculation, it seems that Pro-BNP can be considered an appropriate tool for screening patients with high risk of mortality following sepsis in ED. 

**Table 1:** Baseline characteristics of the studied patients

**Variable **	**Number (%)**
**Sex **	
Male	67 (55.4)
Female	54 (44.6)
**Age (year)**	
20 - 40	3 (2.5)
40 - 60	7 (5.8)
≥ 60	111 (91.7)
**Source of infection**	
Pneumonia	49 (40.5)
Urinary tract infection	45 (37.2)
Digestive problem	14 (11.6)
Bedsore	6 (4.9)
Multiple sources	7 (5.8)
**MEDS score**	
1	21 (17.4)
2	15 (12.4)
3	43 (33.5)
4	15 (12.4)
5	27 (22.3)
**Pro-BNP (pg/ml)**	
< 1000	59 (48.8)
≥ 1000	62 (51.2)

Data are reported as frequency and percentage.

**Table 2:** Screening performance characteristics of Pro-BNP in 1000 pg/ml cut-off and MEDS score in level 3 (moderate to high risk of death)

**Characteristics**	**Pro-BNP**	**MEDS**
**True positive**	59	41
**True negative**	55	57
**False positive**	3	1
**False negative**	4	22
**Sensitivity**	93.6 (83.7-97.9)	65.0 (51.9-76.3)
**Specificity**	94.8 (84.7-98.6)	98.2 (89.5-99.9)
**Positive predictive value**	95.2 (85.6-98.7)	97.6 (85.9-99.8)
**Negative predictive value**	93.2 (82.7-97.8)	72.2 (60.8-81.4)
**Positive likelihood ratio **	19.66 (6.51-59. 3)	41.0 (5.9-284. 4)
**Negative likelihood ratio **	0.07 (0.02-0.18)	0.3 (0.2-0. 5)

Measures are presented with 95% confidence interval.

Presently, various tools have received attention for determining the prognosis of critically ill patients (26, 28, 29). In a study by Varpula et al. in 2007, it was shown that Pro-BNP changes in the first 3 days of hospitalization is a good prognostic scale in septic patients (19). In Kimmoun et al. study in 2013 Pro-BNP level directly correlated with the hemodynamic changes of septic patients (30). A comparison regarding increase in Pro-BNP among cardiac patients and sepsis patients showed a significant increase in the level of this marker in sepsis or septic shock patients compared to cardiac patients (31). In Carpenter et al. study, it was shown that 44% of those with sepsis or septic shock have levels of systolic disorders and in line with this finding they deemed higher serum BNP levels in these patients associated with worse outcome (32). Another study has also suggested the concentration of this biomarker on the 5^th^ day after hospitalization as an index for prognosis of critically ill patients with a higher risk of mortality (33).

On the other hand, accuracy of MEDS score in prediction of 1-month risk of mortality for sepsis patients has been evaluated and declared in numerous studies. Kuo et al. studied 431 patients with pyogenic liver abscesses and affirmed the high accuracy of this scale in this regard (34). Another study in 2003 introduced this scale as a proper tool for triage and making decisions regarding treatment of sepsis patients (10). A study in Netherlands in 2012 indicated the higher power of this scale compared to biomarkers such as CRP and Lactate in prediction of 28-day mortality of sepsis patients in ED (35). Area under the ROC curve of MEDS score in this regard was estimated to be 0.81 in a study by Macdonald et al. (36). 

Considering all the mentioned points as well as some other important points including ease of calculation, availability, higher sensitivity, and similar accuracy and specificity it seems that Pro-BNP can be considered a more beneficial tool compared to MEDS score in prediction of mortality risk and screening of patients with sepsis in ED. It is obvious that doing this screening can help in concentrating the treatment system on more critically ill patients and using more rigorous diagnostic and treatment modalities in trying for improving their outcome.

However, it should be noted that in developing countries such as Iran Pro-BNP measurement is still expensive and it might appear not cost effective to do this evaluation for all sepsis patients. Yet it should be taken into account that proper allocation of funds leads to overall decrease in treatment costs and improvement of patients’ outcome.

***Limitations***

Not differentiating the patients with sepsis, severe sepsis, and septic shock can be one of the limitations of this study. It might have been better to differentiate these patients and evaluate the diagnostic accuracy of the tests in each of the 3 groups to improve the overall accuracy of the study.

## Conclusion:

Based on the findings of the present study, Pro-BNP and MEDS score both have excellent diagnostic accuracy in predicting 1-month mortality of sepsis patients. However, considering the higher sensitivity as well as availability and ease of calculation, it seems that Pro-BNP can be considered an appropriate tool for screening patients with high risk of mortality following sepsis in ED. 
